# Postoperative Cholangitis After Pancreatoduodenectomy: A Frequent Late Complication Demanding Standardized Prevention and Treatment Protocols

**DOI:** 10.1245/s10434-025-18338-x

**Published:** 2025-09-18

**Authors:** Abedin Suljagic, Oskar Swartling, Ebba Seiler Henningson, Christoph Ansorge, Stefan Gilg, Ernesto Sparrelid, Poya Ghorbani

**Affiliations:** 1 Division of Surgery and Oncology, Department of Clinical Sciences, Intervention and Technology, Karolinska Institutet, Stockholm, Sweden; 2 Department of Surgery, Nyköping County Hospital, Nyköping, Sweden; 3 Department of Medicine, Clinical Epidemiology Division, Karolinska Institutet, Stockholm, Sweden; 4 Department of Surgical Sciences, Uppsala University, Uppsala, Sweden; 5 Department of Upper Abdominal Diseases, Karolinska University Hospital, Stockholm, Sweden

**Keywords:** Postoperative cholangitis, Pancreatoduodenectomy, Hepaticojejunostomy, Biliary drainage, Biliary leakage, Morbidity

## Abstract

**Background:**

Postoperative cholangitis (POC), a late complication after pancreatoduodenectomy, is thought to be caused by digestive fluid reflux to the biliary tract or an anastomotic stricture. It is not clear which perioperative risk factors contribute to POC.

**Methods:**

This study included all adult patients undergoing pancreatoduodenectomy between 2008 and 2021 at Karolinska University Hospital, Stockholm, Sweden. Electronic medical records were used to identify patients with POC. Fine and Gray and logistic regression models were used to investigate perioperative risk factors for late cholangitis.

**Results:**

Of the 1002 patients in the study, 86 (9%) experienced POC, and 33 (38% of all the patients with POC) had recurrent POC at least three times during the follow-up period. Preoperative biliary drainage (PBD) was associated with a lower risk of POC (unadjusted subhazard ratio [SHR] 0.60; 95% confidence interval [CI] 0.39–0.92). The patients with Clavien-Dindo grade ≥IIIa complications and those with bile leakage grade B or higher had a higher risk of POC (odds ratio [OR] 1.76 [95% CI 1.11–2.81] and OR 2.76 [95% CI 1.19–6.64], respectively). Intermittent antibiotic treatment was used for 78 (91%) of the POC patients, and 36 (42%) of the POC patients were receiving prophylactic treatment. There were no major differences in the risk of cholangitis and surgical technique, including anastomosis level and suture method.

**Conclusions:**

Postoperative cholangitis after pancreatoduodenectomy is a rather common late complication, occurring in nearly 1 of 10 patients. In this study, preoperative biliary drainage was associated with a decreased risk of cholangitis. Also, the patients with major postoperative complications, including biliary anastomotic leakage, were more likely to experience cholangitis.

Pancreatoduodenectomy (PD) is a high-risk surgical procedure used to treat malignant and benign disorders of the periampullary region and the head of the pancreas. Due to advances in surgical technique and enhanced perioperative care, mortality rates have decreased to below 3% during the last decade, although morbidity rates remain as high as 40% even at high-volume centers.^1,2^

The most frequent short-term complications after PD are postoperative pancreatic fistula (POPF), delayed gastric emptying (DGE), bile leakage, and post-pancreatectomy hemorrhage (PPH). Consequently, these complications have been the subject of attention in numerous publications.^3–5^ However, fewer reports exist on the long-term effects of PD. With the increasing prevalence of diagnosed pancreatic cystic neoplasms (e.g., intraductal papillary mucinous neoplasia), more patients are considered for pre-emptive surgery to avoid cancer transition.^6^ Therefore, more long-term survivors after PD are to be expected.

One of the major late complications is postoperative cholangitis (POC), which occurs in up to 19% of patients after PD and is believed to be caused by anastomotic bile strictures or reflux through the hepaticojejunostomy.^7–10^ Several recent studies have indicated that one of the major risk factor for the development of POC after PD is a small bile duct (<5 mm).^11,12^ Although POC is a common late complication after PD, risk factors, standardized treatment, and prevention protocols are scarce. The primary aims of this study were to examine the prevalence and identify risk factors for cholangitis after PD. The secondary aim was to assess associated morbidity and treatment.

## Methods

This study was a retrospective observational study of patients at least 18 years old undergoing a PD at Karolinska University Hospital Huddinge, a tertiary high-volume referral center for pancreatic surgery, between January 2008 and December 2021. All patients treated with an elective PD for a malignant or benign disease were included, whereas patients who previously underwent a distal pancreatectomy before PD (5 patients) or a previous resection of the extrahepatic bile ducts before PD (2 patients) were excluded. Patients who did not have follow-up data, such as those residing outside the Stockholm region, also were excluded due to limited available follow-up data (207 patients).

All data were prospectively collected and thereafter controlled and classified via computerized patient record systems. The study was approved by the National Ethical Review Agency (registration no. 2020/05238).

### Data Variables and Definition

The criteria for diagnosis of acute cholangitis were first described in 2007 by the Tokyo Guidelines.^13^ In 2018, a second revision of the Tokyo Guidelines (TG18) was established with new standard criteria for the diagnosis of acute cholangitis. The revised guidelines (TG18) consist of three diagnostic categories, each subdivided into clinical symptoms, laboratory data, and radiologic features.^14^

For this study, POC was considered to be present when the TG18 criteria were met or when the clinical diagnosis of cholangitis was reported in the patient’s electronic chart. Recurrent POC was defined as three or more occurrences of postoperative cholangitis using the aforementioned definition.

### Baseline Characteristics

The study collected data on number of patients, age, gender, body mass index (BMI), comorbidities according to the Charlson Comorbidity Index (CCI),^15^ American Society of Anesthesiologists–Physical Status (ASA-PS),^16^ smoking status, administration of neoadjuvant therapy, administration of adjuvant therapy, diagnosis (benign, pre-malignant, malignant), and ECOG/WHO perioperative performance status.^17^ Data on surgical details such as date of surgery, concomitant surgery, artery or vein resection, operation time, type of surgery (laparoscopic, robotic, open, or converted operations), reconstruction type (Modified Child, Roux-en-Y, other), subjective surgical risk assessment of performed hepaticojejunostomy-anastomosis (surgeons’ assessment: low or high), level of performed hepaticojejunostomy anastomosis (common hepatic duct, common bile duct), suture technique (interrupted, running, mixed, other), and suture caliber size (4-0 to 6-0) were collected.

### Preoperative Variables

The study collected data on preoperative obstructive jaundice (yes/no), preoperative biliary drainage (PBD) (yes/no), and preoperative cholangitis (yes/no). A predetermined cutoff bilirubin level for performing PBD was not available for this study period.

### Perioperative Variables

The patients underwent either a pylorus-preserving pancreatoduodenectomy (PPPD) or a conventional PD (Whipple). Most reconstructions were performed using the modified Child method with or without Braun’s anastomosis.^18^ Pancreatojejunostomy was performed using the two-layer duct-to-mucosa method in most cases.^19^ Transection of the bile duct was made above the junction of the cystic duct and the common hepatic duct in every case, but the level of performed hepaticojejunostomy varied depending on the size and thickness of the bile duct as well as the surgeon’s preference. Varied suture techniques including interrupted, running, and combined sutures were used depending on the individual anastomosis need for tension and strength. Suture size also varied from absorbable 4-0 to 6-0 caliber sutures. Absorbable sutures were used almost exclusively. Also, data on perioperative obstructive jaundice were collected (yes/no).

### Postoperative Variables

The study collected data on length of postoperative intensive care unit (ICU) stay, complications according to Clavien-Dindo classification,^20^ date of discharge, discharge destination, length of hospital stay, date of death, delayed gastric emptying (DGE), post-pancreatectomy hemorrhage (PPH), postoperative pancreatic fistula (POPF) according to International Study Group of Pancreatic Surgery (ISGPS), and bile leakage according to International Study Group of Liver Surgery (ISGLS).^4,21–23^ Only grade B or C was considered to be clinically relevant. Also, histologic diagnosis and pathologic staging according to TNM7 classification were collected,^24^ as well as data on number of POC episodes, readmission, intermittent or prophylactic antibiotic treatment, liver abscess, sepsis, multi-organ failure, and mortality (all associated to POC) were collected.

### Statistical Analysis

Values for continuous data were calculated using median and interquartile range (IQR) or mean and standard deviation and analyzed using the Student’s *t* test. Categorical data were analyzed with chi-square or Fisher’s exact test, when appropriate, and expressed as absolute values and proportions. Pre- and intraoperative risk factors for POC were analyzed using Fine and Gray models with subhazard ratios (SHRs) and 95% confidence intervals to account for death as a competing event.

Patients were considered at risk until POC (outcome), death (competing event), or last follow-up evaluation, whichever happened first. The models then were adjusted for age, sex, BMI, smoking status (never, current, previous), and Charlson Comorbidity Index.^15^ Risk factors that can develop during the postoperative course were analyzed using logistic regression models and presented as odds ratios (ORs) and 95% confidence intervals (CIs). These models then were adjusted for the aforementioned variables.

In a secondary analysis, logistic regression models were used to analyze the odds of recurrent cholangitis episodes, defined as three or more cholangitis occasions. Kaplan-Meier failure estimates were used to visualize the time to the first episode of postoperative cholangitis among affected patients. Analyses were performed using Stata version 16 software (Stata Corp, College Station, TX, USA).

## Results

### Baseline Characteristics

The study enrolled 1002 patients treated with PD. Of these patients, 46% were women. The overall median age was 69 years (IQR 61–75 years; Table 1)*.* Comorbidities were common, with 375 (37%) cases of hypertension, 196 (20%) cases of diabetes, and 31 (12%) cases of cerebrovascular disease before surgery. A majority of the surgically treated patients had an ASA score of 2 (555 patients, 55%) and a median Charlson Comorbidity Index of 5 (IQR 4–6). The median follow-up time after surgery was 2.5 years (IQR 1.3–4.0 years), with a mean of 3.1 ± 2.6 years. Postoperative histologic examination showed that 788 (79%) of all the patients underwent surgery due to a malignant disease.

**Table 1:** Patient characteristics

	Postoperative cholangitis			Overall *n* (%)
	No cholangitis *n* (%)	Cholangitis *n* (%)	*p* Value	
No. of patients	916 (91.4)	86 (8.6)		1002 (100)
Median age: years (IQR)	69.0 (61.5–74.8)	66.9 (61.0–73.3)	0.109	68.9 (61.4–74.8)
Male sex	488 (53.3)	51 (59.3)	0.284	539 (53.8)
Mean BMI (kg/m^2^)	25.1 ± 4.7	25.6 ± 3.6	0.253	25.1 ± 4.6
Comorbidities				
Hypertension	351 (38.3)	24 (27.9)	0.056	375 (37.4)
Diabetes	178 (19.5)	18 (21.0)	0.741	196 (19.6)
Cerebrovascular disease	28 (12.6)	3 (10.7)	0.780	31 (12.4)
Smoking			0.332	
No	621 (67.9)	59 (68.6)		680 (67.9)
Yes	153 (16.7)	10 (11.6)		163 (16.3)
Previous	141 (15.4)	17 (19.8)		158 (15.8)
ASA score			0.299	
1	94 (10.3)	12 (14.0)		106 (10.6)
2	507 (55.4)	48 (55.8)		555 (55.4)
3	300 (32.8)	23 (26.7)		323 (32.3)
4	14 (1.5)	3 (3.5)		17 (1.7)
Median CCI (IQR)	5 (4–6)	5 (4–6)	1.00	5 (4–6)
Neoadjuvant treatment	39 (4.4)	4 (5.0)	0.814	43 (4.5)
Adjuvant treatment			0.673	
No	712 (77.7)	66 (76.7)		778 (77.6)
Yes	123 (13.4)	14 (16.3)		137 (13.7)
Partially	81 (8.8)	6 (7.0)		87 (8.7)
Diagnosis			0.002	
Benign	72 (7.9)	10 (11.6)		82 (8.2)
Malignant	733 (80.0)	55 (64.0)		788 (78.6)
Pre-malignant/other	111 (12.1)	21 (24.4)		132 (13.2)

Values are mean ± standard deviation for continuous data or median (IQR) and count (%) for categorical data. Analyzed with the chi-square test or Fisher’s exact test for categorical variables and with Student’s *t* test for continuous variables.

*IQR* interquartile range, *BMI* body mass index, *ASA* American Society of Anesthesiologists’ classification system, *CCI* Charlson Comorbidity Index

### Postoperative Cholangitis

During the follow-up period, 86 (9%) of the patients experienced at least one episode of POC. Of these patients, 41 (48%) experienced POC during the first year after surgery. The median time to the first POC was 1 year (IQR, 0.5–1.9 years), with a mean time of 1.6 ± 1.9 years.

**Fig. 1:**
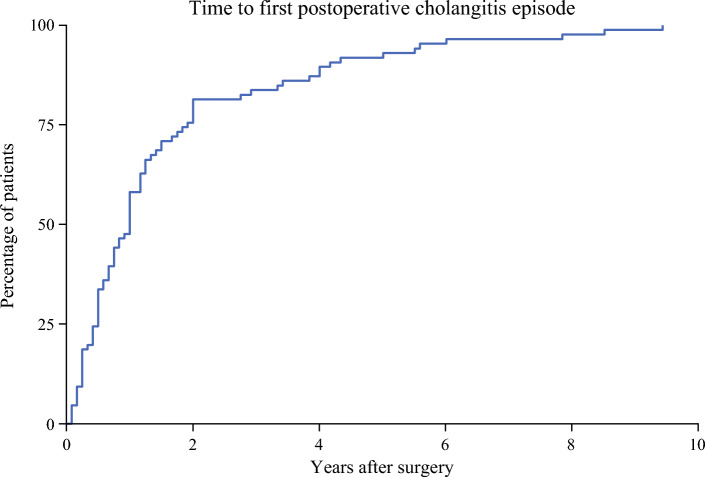
Kaplan-Meier graph of time to first episode of postoperative cholangitis among patients that developed postoperative cholangitis

The patients with benign or pre-malign disease were more likely to experience POC (Table 1). Two episodes of POC were experienced by 61 patients (71% of all the patients with POC), and 33 patients (38% of all the patients with POC) had more than three episodes of POC. Figure 1 depicts the Kaplan-Meier failure estimate of time to the first POC among the affected patients.

### Pre- and Intraoperative Risk Factors for Postoperative Cholangitis

Few differences in patient characteristics and risk of POC after PD were observed, and no increased risk of POC was based on age, sex, BMI, or preoperative comorbidities (Table 2). However, the patients with a wider bile duct due to preoperative obstructive jaundice had a lower risk of POC (unadjusted SHR 0.65; 95% CI 0.43–0.99; *p* = 0.047; adjusted SHR 0.65; 95% CI 0.42–0.99; *p* = 0.049) than the patients without obstructive jaundice. Furthermore, the patients undergoing preoperative biliary draining or stenting had a lower SHR for POC (unadjusted SHR 0.60; 95% CI 0.39–0.92; *p* = 0.018; adjusted SHR 0.60; 95% CI 0.39–0.91; *p* = 0.018). The patients with preoperative obstructive jaundice also had decreased odds of recurrent cholangitis episodes (unadjusted OR 0.50; 95% CI 0.27–0.92; Table 3). The patients with preoperative cholangitis had no greater risk of POC. Also, there were few differences in intraoperative characteristics and risk of later POC (Table 4).

**Table 2:** Preoperative characteristics and risk for postoperative cholangitis

	SHR (95% CI) for postoperative cholangitis^a^			
	Unadjusted	*p* Value	Adjusted^b^	*p* Value
Age (years)	0.99 (0.97–1.01)	0.229	0.99 (0.96–1.01)	0.299
Sex: men vs women	1.26 (0.82–1.94)	0.290	1.25 (0.81–1.92)	0.305
BMI (kg/m^2^)	1.02 (0.99–1.05)	0.238	1.01 (0.98–1.04)	0.470
Comorbidities				
Hypertension	0.66 (0.41–1.05)	0.078	0.58 (0.24–1.00)	0.051
Diabetes	1.10 (0.66–1.85)	0.718	1.20 (0.61–1.99)	0.746
Cerebrovascular disease	0.83 (0.26–2.71)	0.763	0.83 (0.27–2.52)	0.741
Smoking				
No	1.00 (Reference)		1.00 (Reference)	
Yes	0.69 (0.36–1.35)	0.281	0.66 (0.34–1.29)	0.223
Previous	1.37 (0.80–2.36)	0.251	1.40 (0.81–2.41)	0.228
ASA score				
1	1.00 (Reference)		1.00 (Reference)	
2	0.78 (0.42–1.45)	0.432	0.83 (0.46–1.52)	0.553
3	0.65 (0.33–1.29)	0.219	0.68 (0.34–1.37)	0.287
4	1.61 (0.49–5.28)	0.435	1.55 (0.44–5.41)	0.491
CCI	0.96 (0.82–1.12)	0.598	1.01 (0.82–1.25)	0.913
Neoadjuvant treatment	1.14 (0.42–3.11)	0.801	1.07 (0.38–2.98)	0.898
Preoperative obstructive jaundice	0.65 (0.43–0.99)	0.047	0.65 (0.42–0.99)	0.049
Preoperative biliary drainage and/or stent	0.60 (0.39–0.92)	0.018	0.60 (0.39–0.91)	0.018
Preoperative cholangitis	1.43 (0.69–2.98)	0.334	1.43 (0.68–3.01)	0.352

*SHR* subhazard ratio, *CI* confidence interval, *BMI* body mass index, *ASA* American Society of Anesthesiologists’ classification system, *CCI* Charlson Comorbidity Index

^a^Subhazard ratios for postoperative cholangitis

^b^Adjusted for age, sex, BMI, smoking status and Charlson comorbidity index

**Table 3:** Risk factors for multiple cholangitis episodes after pancreatoduodenectomy

	OR (95% CI) of postoperative cholangitis				Recurrent cholangitis episodes			
	Unadjusted OR	*p* Value	Adjusted OR^a^	*p* Value	Unadjusted OR	*p* Value	Adjusted OR*	*p* Value
Preoperative obstructive jaundice	0.60 (0.39–0.92)	0.020	0.59 (0.38–0.91)	0.018	0.50 (0.27–0.92)	0.027	0.50 (0.27–0.93)	0.028
Preoperative biliary drainage and/or stent	0.56 (0.36–0.86)	0.009	0.55 (0.36–0.86)	0.009	0.54 (0.29–1.01)	0.053	0.55 (0.29–1.02)	0.059
Perioperative obstructive jaundice	0.63 (0.33–1.20)	0.159	0.60 (0.31–1.16)	0.128	0.21 (0.05–0.86)	0.030	0.19 (0.46–0.80)	0.024
Clavien-Dindo ≥IIIa	1.65 (1.04–2.60)	0.033	1.63 (1.03–2.59)	0.038	1.30 (0.67–2.53)	0.443	1.28 (0.65–2.51)	0.471
ICU admission	1.17 (0.49–2.29)	0.720	1.16 (0.48–2.78)	0.741	0.42 (0.06–3.07)	0.390	0.39 (0.05–2.94)	0.364
Bile leakage ≥B	2.76 (1.19–6.44)	0.019	2.54 (1.08–5.99)	0.033	2.17 (0.64–7.40)	0.214	1.97 (0.57–6.82)	0.283

*OR* odds ratio, *CI* confidence interval, *ICU* intensive care unit

^a^Adjusted for age, sex, BMI, smoking status, and Charlson Comorbidity Index

**Table 4:** Intraoperative risk factors for postoperative cholangitis after pancreatoduodenectomy

	*n* (%) of patients		SHR (95% CI) for postoperative cholangitis^a^			
	No POC	POC	Unadjusted SHR	*p* Value	Adjusted SHR^b^	*p* Value
Perioperative obstructive jaundice	173 (19.0)	11 (12.9)	0.66 (0.35–1.25)	0.201	0.65 (0.34–1.24)	0.193
Operation time ≥390 min	576 (62.9)	58 (67.4)	0.29 (0.80–1.95)	0.336	1.21 (0.77–1.89)	0.415
Artery resection (hepatic)	16 (1.9)	2 (2.6)	1.28 (0.32–5.02)	0.728	1.31 (0.34–5.03)	0.696
Vein resection	177 (19.5)	21 (24.4)	1.32 (0.81–2.15)	0.268	1.36 (0.83–2.23)	0.229
Reconstruction						
Modified Child	892 (97.4)	84 (97.7)	1.00 (Reference)		1.00 (Reference)	
Roux-en-Y	12 (1.3)	1 (1.2)	0.89 (0.12–6.53)	0.907	0.83 (0.11–6.30)	0.860
Other	12 (1.3)	1 (1.2)	0.90 (0.12–6.65)	0.919	0.90 (0.12–6.83)	0.921
Anastomosis level						
CHD	529 (59.4)	48 (55.8)	1.00 (Reference)		1.00 (Reference)	
CBD	361 (40.6)	38 (44.2)	1.09 (0.72–1.66)	0.689	1.13 (0.74–1.72)	0.567
Anastomosis suture technique						
5.0 Interrupted	763 (84.5)	66 (78.6)	1.00 (Reference)		1.00 (Reference)	
5.0 Running	14 (1.6)	1 (1.2)	0.86 (0.12–6.32)	0.882	0.85 (0.11–6.61)	0.880
5.0 Mix	3 (0.3)	2 (2.4)	7.24 (1.76–29.80)	0.006	7.34 (1.68–31.99)	0.008
6.0 Interrupted	94 (10.4)	12 (14.3)	1.41 (0.77–2.59)	0.261	1.40 (0.77–2.54)	0.275
6.0 Mix	5 (0.6)	0 (0)	N/A		N/A	
Other	24 (2.7)	3 (3.6)	1.52 (0.47–4.90)	0.480	1.54 (0.47–5.02)	0.477
Anastomosis risk						
Low	19 (2.1)	4 (4.7)	1.00 (Reference)		1.00 (Reference)	
High	896 (97.9)	82 (95.4)	2.26 (0.83–6.21)	0.112	2.28 (0.79–6.58)	0.129

*SHR* subhazard ratio, *CI* confidence interval, *POC* postoperative cholangitis, *CHD* common hepatic duct, *CBD* common bile duct

^a^Subhazard ratios for postoperative cholangitis

^b^Adjusted for age, sex, BMI, smoking status and Charlson Comorbidity Index

### Postoperative Risk Factors for Cholangitis

The patients with Clavien-Dindo ≥IIIa had a greater risk of POC than the patients with a lower Clavien-Dindo grade (unadjusted OR 1.76; 95% CI 1.11–2.81; *p* = 0.017; adjusted OR 1.76; 95% CI 1.10–2.81; *p* = 0.018). Also, the patients who had grade B or higher bile leakage had greater odds of POC (unadjusted OR 2.81; 95% CI 1.19–6.64; *p* = 0.018; adjusted OR 2.63; 95% CI 1.10–6.27; *p* = 0.029). There was no significantly increased risk of POC depending on hospital length of stay, intensive care unit (ICU) admission status, or other pancreas-specific postoperative complications (Table 5).

**Table 5:** Postoperative complications and cholangitis after pancreatoduodenectomy

Postoperative complication	Postoperative cholangitis vs no cholangitis			
	Unadjusted OR (95% CI)	*p* Value	Adjusted OR^a^ (95% CI)	*p* Value
Clavien-Dindo ≥IIIa	1.76 (1.11–2.81)	0.017	1.76 (1.10–2.81)	0.018
ICU admission	1.39 (0.58–3.43)	0.467	1.39 (0.57–3.38)	0.467
Hospital stay ≥14 days	1.03 (0.66–1.61)	0.899	1.06 (0.67–1.67)	0.795
DGE ≥B	1.06 (0.65–1.71)	0.816	1.10 (0.67–1.79)	0.710
POPF ≥B	1.44 (0.85–2.45)	0.174	1.36 (0.79–2.32)	0.268
PPH ≥B	1.16 (0.60–2.25)	0.666	1.14 (0.58–2.22)	0.709
Bile leakage ≥B	2.81 (1.19–6.64)	0.018	2.63 (1.10–6.27)	0.029

*OR* odds ratio, *CI* confidence interval, *ICU* intensive care unit, *DGE* delayed gastric emptying, *POPF* postoperative pancreatic fistula, *PPH* post-pancreatectomy hemorrhage.

Unadjusted and adjusted logistic regression of postoperative cholangitis and complications after pancreatoduodenectomy

^a^Adjusted for age, sex, BMI, smoking status and Charlson Comorbidity Index

### Morbidity Associated to POC

During the follow-up period, 24 patients experienced sepsis, and 6 patients experienced multiorgan failure due to POC. Moreover, 19 patients with POC later experienced liver abscesses during follow-up evaluation. Intermittent antibiotic treatment was observed in 78 patients, and 36 patients had long-term prophylactic antibiotic treatment. The treatment for 26 patients (30% of all the patients with POC) was endoscopy.

The documented treatment attempts included endoscopic balloon dilation of the hepaticojejunostomy, endoscopic balloon dilation with stent placement, and endoscopic stent placement alone. Successful endoscopic treatment response was observed in two patients who received a stent only, in one patient treated with balloon dilation and a stent, and in one patient treated with endoscopic stone extraction.

Endoscopic treatment attempts were unsuccessful for 12 patients, mostly due to technical difficulties reaching the hepaticojejunostomy. Nine of these patients received a complementary biliary drainage with percutaneous transhepatic cholangiography (PTC), and one patient was treated with PTC alone.

Documented PTC-related interventions included one balloon dilation and one balloon dilation with stent placement. Three patients received a stent via PTC, and three patients were treated with external PTC drainage alone. For 8 (80%) of 10 patients, treatment with PTC was successful. Five of the patients treated with endoscopy, radiologic intervention, or both showed no visible strictures during the attempted procedure.

Four patients (5% of the POC patients) were treated with surgical reconstruction of the hepaticojejunostomy due to recurrent POC and insufficient endoscopic, radiologic, and antibiotic treatment response. Three of these patients experienced a permanent alleviation of cholangitis with no additional POC episodes. No POC-related mortality was observed.

## Discussion

This observational study from a large European high-volume pancreatic center including 1002 patients who underwent PD from 2008 to 2021 found that 86 (9%) patients experienced POC during the postoperative course. The risk factors for the development of POC were major postoperative complications, such as Clavien-Dindo ≥IIIa and bile leakage grade ≥B. Preoperative obstructive jaundice and preoperative biliary drainage were found to be associated with a decreased risk for the development of POC after PD, presumably due to obstruction, enlargement, and thickening of the bile duct. This also was found in patients with recurrent cholangitis episodes. Interestingly, preoperative cholangitis was not associated with an increased risk for the development of POC. Furthermore, few differences in intraoperative characteristics and risk for POC where observed. Recent publications on the subject have shed new light on POC-associated morbidity, in conjunction with previous literature, implicating the need for an agile treatment response.^10,25,26^

The incidence of POC in our study showed that 86 (9%) of the included patients experienced at least one episode of POC, with 41 patients (48%) of those affected experiencing POC during the first year after surgery. Also, we identified 61 patients (71% of all the patients with POC) who experienced more than one cholangitis episode, and 33 patients (38% of all the patients with POC and 3% of all the PDs) had more than three episodes of POC. This can be compared with a Japanese study reporting that 19% of patients undergoing PD experienced recurrent POC, defined as three or more cholangitis episodes.^10^ The difference in incidence of POC between our study and the study of Ueda et al.^10^ also could have been due to their higher percentage (35%) of patients undergoing PD for a pre-malignant and benign diagnosis who had a non-dilated common bile duct.

According to our findings, the patients with preoperative obstructive jaundice and those who underwent preoperative biliary drainage or stenting had a decreased risk for the development of POC or recurrent POC episodes. This accords with a recent study suggesting that non-biliary drainage is a risk factor for the development of late-onset POC after PD.^27^ However, the potential benefit of PBD in this setting should be seen in light of its associated preoperative risk (i.e., complications such a pancreatitis that can delay the time to surgery and worsen the perioperative circumstances due to scar tissue formation).^12^

Several retrospective studies have indicated that benign disease is a risk factor for POC due to surgeries performed on non-dilated bile ducts.^10,12^ However this also could be explained by a longer survival among patients with benign disease, which is why we used competing risk-adjusted models in the current study.

Biliary leakage after PD is relatively rare, with complication severity ranging from trivial to life-threatening. Surgical intervention is rarely required, and most leakages can be managed safely with percutaneous transhepatic biliary drainage.^28,29^ In addition to bile leakage, this study found that major complications (Clavien-Dindo >III) resulted in increased risk for the development of POC. Although we cannot rule out the possibility that bile leakage and major complications may separately contribute to POC, it is plausible that bile leakage occurs due to impaired healing processes as a consequence of major complications.

The pathophysiology of cholangitis after PD remains unknown, with several mechanisms suggested, possibly in association, such as biliary stricture, delayed gastric emptying or ileus and obstruction by alimentary debris, or bile contamination by germs resistant to antimicrobial prophylaxis.^12^ House et al.^8^ investigated the risk of biliary strictures in patients undergoing PD with postoperative bile leakage and found that only 5% of patients later found to have biliary strictures after PD had presented with postoperative bile leakage. This indicates that bile leakage may not be an indicator for the development of biliary strictures and POC, contradicting our finding that clinically significant bile leakage was associated with a higher risk of POC. In a recent study by Henry et al.^25^ with a similar cohort size and a comparable incidence of 10 % POC after PD, biliary leakage was reported as a significant risk factor for the development of POC.

In our study, we identified the need for intermittent antibiotic treatment for 78 patients, and 36 patients had long-term prophylactic antibiotic treatment. Several patients received both intermittent and prophylactic antibiotic treatment, probably due to the occurrence of recurrent cholangitis. Camman et al.^30^ demonstrated that the risk for the presence of drug-resistant bacteria is increased by preoperative stenting of the common bile duct. They found that patients were more likely to experience strains with resistance against the antibiotic given intraoperatively. Our patients received a generic predestined antibiotic compound preoperatively. All perioperatively harvested PBD stent/drainages were sent for culture analysis and thereafter were treated accordingly when clinically indicated. Although a more common treatment for primary biliary cholangitis, ursodeoxycholic acid also may be used as treatment for POC. Ursodeoxycholic acid is a gallstone dissolvent with an anti-inflammatory effect.^31^ It often is used clinically as a treatment option in our clinic and recently is recommended as part of a treatment regimen for non-obstructive postoperative cholangitis.^26^

Of all the cholangitis patients, 30% were treated with endoscopy for POC due to suspected biliary stricture. Only four patients experienced a successful outcome, and nine patients (39%) received complementary radiologic intervention treatment (PTC). These numbers question endoscopy as a reliable treatment option for biliary strictures, especially considering the technical difficulties reaching the hepaticojejunostomy with a double-balloon technique and the recurrent nature of a biliary stricture. According to House et al.,^8^ 7.5 interventions were required to achieve complete alleviation of the condition. The incidence of biliary strictures after PD ranges from 3 to 7%, and most cases of cholangitis are considered to result from biliary strictures.^8,9,32^

In our study, 2% of the entire cohort but only 24% of all the POC patients had a diagnosis of biliary strictures. This accords with the aforementioned results and also is in line with a previous study implying that POC is known to occur even in the absence of intrahepatic biliary duct dilation.^33^ A previous study described surgical innovations of the hepaticojejunostomy performed on small-diameter bile ducts with an improved POC outcome after PD.^34^ Also, a newly published study describing a novel hepaticojejunostomy technique with a larger anastomotic diameter is showing promising results with a significant reduction of POC after PD.^35^

**Fig. 2:**
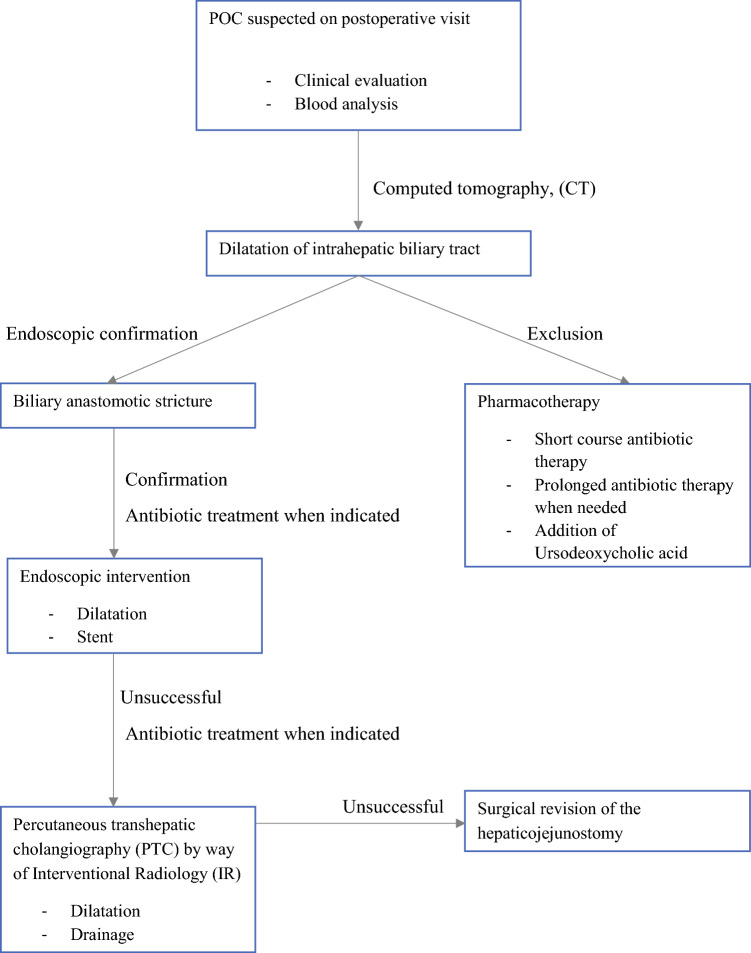
Treatment protocol algorithm for patients with postoperative cholangitis after pancreatoduodenectomy.

Prevention of POC is challenging, and technical difficulties performing anastomoses on bile ducts with a small diameter remain, Early diagnostics and treatment protocols currently seem more feasible. Figure 2 presents a flowchart for the management of POC, with a focus on early diagnosis (Fig. 2).^25,26^

One limitation of this study was its retrospective nature, which limited the ability to shape the cohort according to preselected variables and to establish specific follow-up routines. Also, the diagnosis of POC was based on either the TG18 criteria or the time when the clinical diagnosis of cholangitis was reported in the patient’s electronic chart, which could inflict a registration bias and reduced comparability with previous literature.

Our study also had strengths. One strength was the large study population in conjunction with the fact that all the patients underwent the same type of resection. Another strength was the long follow-up time.

## Conclusions

Postoperative cholangitis after pancreatoduodenectomy occurred for 9% of all the patients who underwent surgery, and preoperative biliary drainage was associated with a decreased risk of cholangitis. Also, the patients with major postoperative complications, including biliary anastomotic leakage, were more likely to experience cholangitis. Postoperative cholangitis occurs frequently and does not lead to severe morbidity or mortality, but presumably imposes a reduction in quality of life due to its recurrent nature. Early assertive and targeted therapeutic protocols are needed to improve clinical outcomes for this patient category.

## Data Availability

Data, analytic methods, and study materials are available from the corresponding author upon reasonable request. The authors had complete access to the study data that support this article. The research has not been preregistered.
